# Association between the neutrophil‐to‐lymphocyte ratio and intravesical prostatic protrusion in men with benign prostatic hyperplasia

**DOI:** 10.1111/luts.12287

**Published:** 2019-09-15

**Authors:** Mun Su Chung, Yun Jung Yang, Seung Hwan Lee, Byung Il Yoon

**Affiliations:** ^1^ Department of Urology International St. Mary's Hospital, Catholic Kwandong University Incheon South Korea; ^2^ Institute of Biomedical Science International St. Mary's Hospital, Catholic Kwandong University Incheon South Korea; ^3^ Department of Urology Urological Science Institute, Yonsei University College of Medicine Seoul South Korea

**Keywords:** inflammation, intravesical protrusion, neutrophil‐to‐lymphocyte ratio, prostate

## Abstract

**Objective:**

To analyze the association between neutrophil‐to‐lymphocyte ratio (NLR) and intravesical prostatic protrusion (IPP) in men with benign prostatic hyperplasia.

**Methods:**

Two hundred and fifty men aged >50 years who presented with lower urinary tract symptoms at our institution between 2014 and 2018 were analyzed. Pearson's method was used for analysis of the correlation between NLR and IPP. Multivariate logistic regression analysis was used to identify predictors of IPP. Further analysis according to total prostate volume (TPV) was performed.

**Results:**

The NLR correlated positively with IPP (Pearson's r = 0.459, *P* < 0.001) and was an independent predictor of IPP ≥10 mm (odds ratio, 2.95; 95% confidence interval, 1.59–5.47; *P* = 0.0006). Among the 142 men with prostates <40 cm^3^, mean NLR was 2.50 ± 0.71 in those with IPP ≥10 mm and 1.71 ± 0.57 in those with IPP < 10 mm (*P* < 0.001). The NLR differed significantly between those with a prostate <40 cm^3^ and IPP ≥10 mm and those with a larger prostate and IPP < 10 mm (2.50 ± 0.71 vs 2.07 ± 0.77, respectively; *P* = 0.020).

**Conclusions:**

NLR can be used as a surrogate marker for presence of IPP. Its clinical value would be especially important in men with a small prostate gland but high IPP. The NLR seemed to be more strongly correlated with IPP than with TPV.

## 1 INTRODUCTION

Male lower urinary tract symptoms (LUTS) have conventionally been considered as merely the result of age‐related prostatic enlargement. However, such a simple explanation is not accepted due to the heterogenous characteristics of LUTS and their relationships with systemic diseases.^1^

Numerous studies have demonstrated that benign prostatic hyperplasia (BPH) may be caused by a chronic inflammatory process or immune cell infiltration.^2^, ^3^, ^4^, ^5^, ^6^ In response to prostatic inflammation, immune cells generate cytokines that affect other cells to produce growth factors. This enhances the proliferation of stromal and epithelial cells, and this is sustained by an autoimmune mechanism, leading to an increase in prostate volume.^5^ Chronic inflammation can cause tissue damage, potentially resulting in a repetitive process of wound healing which is associated with BPH.^3^, ^4^, ^5^, ^6^

Recently, the neutrophil‐to‐lymphocyte ratio (NLR) has been proposed as a surrogate marker for various conditions, including the systemic inflammatory response, metabolic syndrome, outcomes in oncologic fields, cardiovascular disorders, and other medical disorders.^7^, ^8^, ^9^, ^10^, ^11^, ^12^, ^13^ There have also been reports of its significance in urology. For example, associations have been reported with biochemical failure in prostate cancer,^14^ the cancer‐specific survival of patients with metastatic renal cell carcinoma,^15^ and the spontaneous passage of ureteral stones.^16^ Ozer et al.^3^ showed an association of NLR with severe LUTS and the progression of BPH. Similarly, Tanik et al.^2^ suggested NLR was a predictor of BPH progression. However, there are only limited data on an association between NLR and intravesical prostatic protrusion (IPP), an anatomical feature caused by the growth of prostatic lateral and median lobes. Considering the role of IPP as a marker of bladder outlet obstruction (BOO),^17^, ^18^, ^19^, ^20^ we need well‐designed studies to establish how prostatic morphological features (especially the degree of IPP, as well as the total prostate volume [TPV] and the transitional zone volume [TZV]) vary according to the NLR. We aimed to analyze the association between NLR and the degree of IPP. To our knowledge, this is the first study on this topic.

## 2 METHODS

### 2.1 Ethics statement

This study was approved by the Institutional Ethics Committee of Catholic Kwandong University College of Medicine after reviewing the study protocol and procedures (IS18RISI0076). The requirement for written consent was waived because of the retrospective nature of the study. The data were anonymized before the analysis.

### 2.2 Study population

Medical records from 250 men aged >50 years who presenting with LUTS at our outpatient clinic between January 2014 and December 2018 were retrospectively analyzed. We collected data on patient demographics and clinical characteristics, including age, body mass index, prostate‐specific antigen values, TPV and TZV on transrectal ultrasound (TRUS), degree of IPP, and neutrophil/lymphocyte counts from peripheral blood samples. The peripheral blood sampling for NLR assessment was done just before performing TRUS. The exclusion criteria included the following: suspected bacterial or viral infection by laboratory results; malignancy; autoimmune or systemic inflammatory diseases that may influence NLR values; the use of anti‐inflammatory drugs or 5‐alpha reductase inhibitors; immunotherapy; urinary tract stone; and history of prostatic surgery. Patients with incomplete data were excluded from the statistical analysis.

### 2.3 Measurement of the prostate

TPV, TZV, and IPP were measured using TRUS. TPV was automatically calculated by multiplying together the largest antero‐posterior, transverse, and cephalocaudal diameters, and multiplying this by 0.52. Measuring TZ was done in a similar manner, as previously presented.^21^ Determination of IPP was done after checking the vertical length between two points; end of the protrusion and base of bladder.^18^ All the measurements were made by a single urologist (MSC).

### 2.4 Statistical analysis

To compare continuous and categorical variables, Student's *t* test (or Kruskal–Wallis test) and the χ^2^ test were performed. Pearson's method was used for the correlation analysis. Multivariate logistic regression models that included all the collected variables were constructed to identify the factors that were predictive of IPP. The cut‐off values were determined using the area under the receiver operating characteristic (ROC) curve. The statistical analyses were performed with R statistics version 3.5.1. Results were considered significant at *P* < 0.05.

## 3 RESULTS

**Figure 1:**
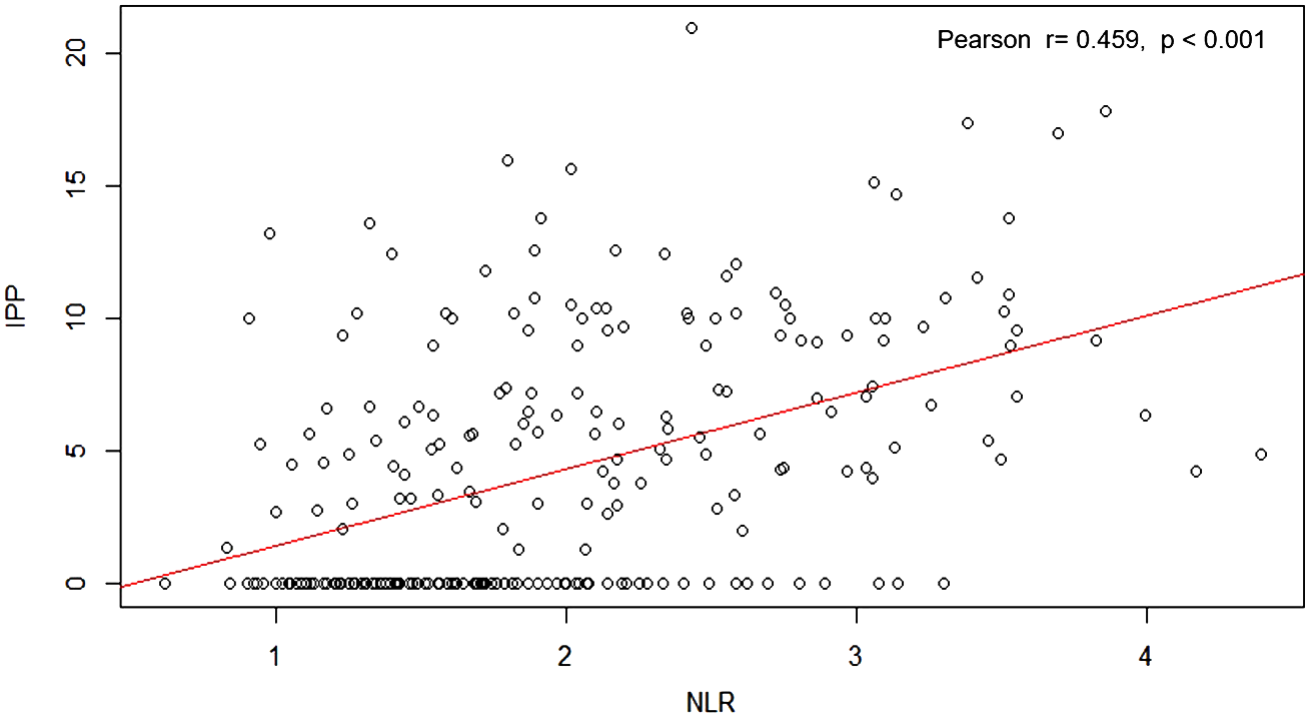
Correlation between the neutrophil‐to‐lymphocyte ratio (NLR) and intravesical prostatic protrusion (IPP) [Colour figure can be viewed at http://wileyonlinelibrary.com]

**Figure 2:**
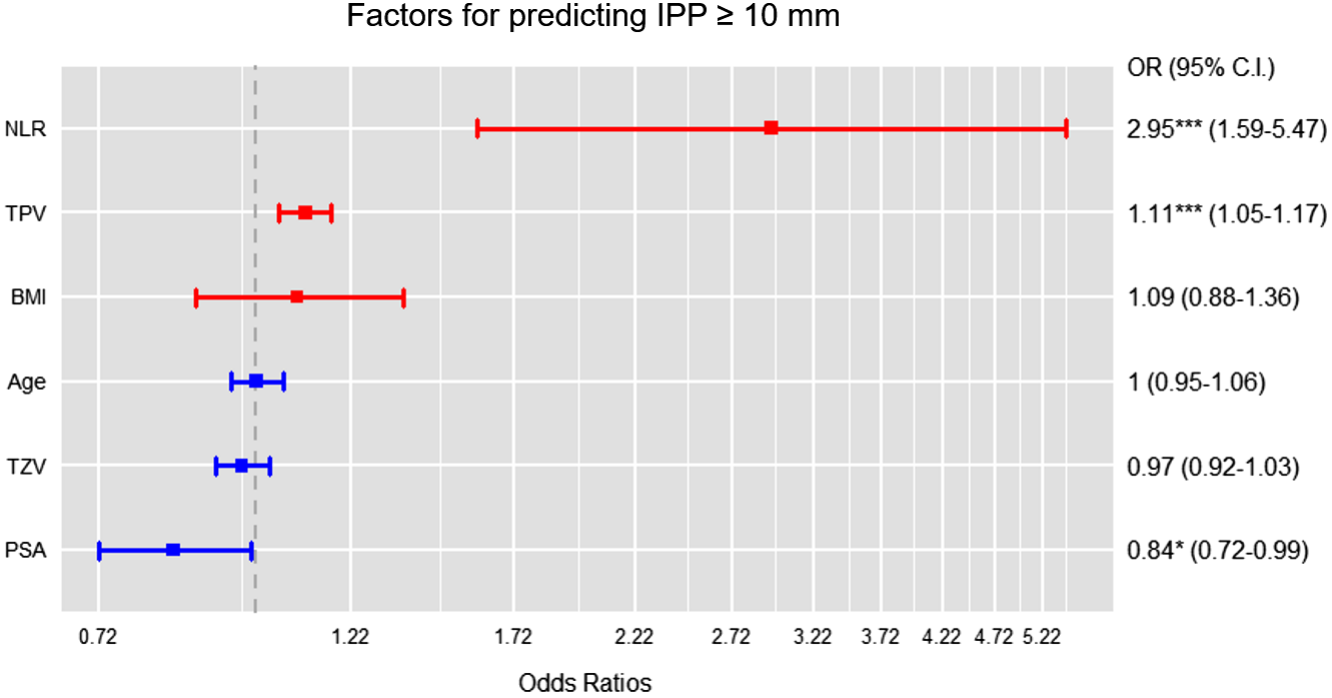
Multivariate logistic regression analysis in the prediction of intravesical prostatic protrusion (IPP) ≥10 mm. *P < 0.05, ***P < 0.001 [Colour figure can be viewed at http://wileyonlinelibrary.com]

**Figure 3:**
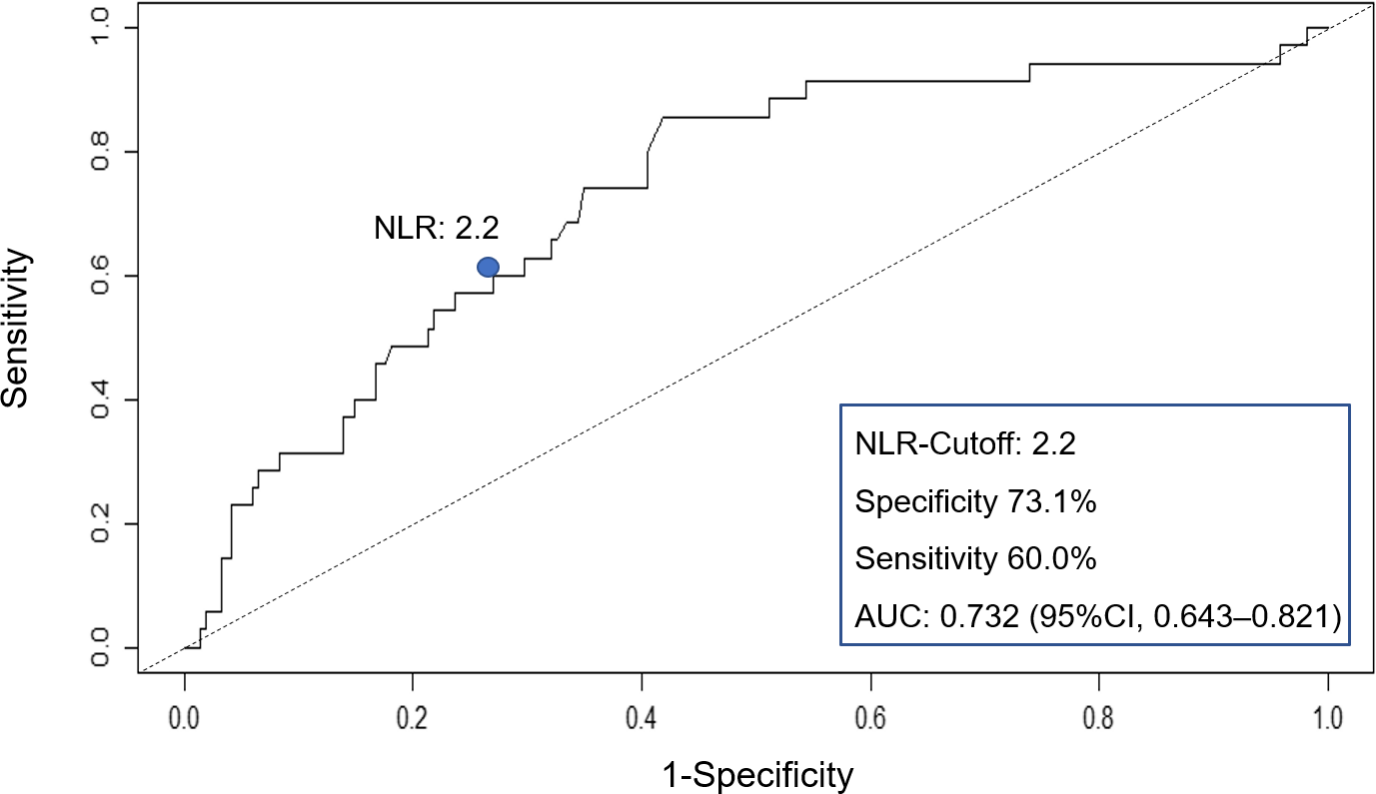
Receiver operating characteristic (ROC) curve to predict intravesical prostatic protrusion (IPP) ≥10 mm [Colour figure can be viewed at http://wileyonlinelibrary.com]

Table 1 summarizes the baseline patient characteristics. The median IPP was 3.3 mm (interquartile range, 0.0–7.4), and 24.4% (61/250) of the patients had IPP ≥10 mm. In the overall cohort, there was a significant difference in NLR according to IPP (*P* < 0.001, Table 2). Pearson correlation analysis revealed a positive correlation between NLR and IPP (*P* < 0.001, r = 0.459; Figure 1). The predictors for IPP ≥10 mm are presented in Figure 2. In the multivariate logistic regression analysis, NLR was found to be an independent predictor of IPP ≥10 mm (odds ratio [OR] 2.95; 95% confidence interval [CI] 1.59–5.47; *P* = 0.0006). TZV was not a significant predictor of IPP (OR 0.97; 95% CI 0.92–1.03; *P* = 0.341). The area under the curve obtained from the ROC curve was 0.732 (95% CI: 0.643–0.821). The NLR value with the highest combined sensitivity and specificity predicting IPP ≥10 mm in our cohort was 2.2 (specificity: 73.1%, sensitivity: 60.0%; Figure 3).

**Table 1:** Baseline characteristics of patients (n = 250)

Median (interquartile range)	
Age, y	65.0 (57.5–71.0)
BMI, kg/m^2^	24.1 (22.6–25.6)
PSA, ng/mL	1.8 (0.9–3.4)
Prostate volume on TRUS, cm^3^	
TPV	39.5 (24.0–61.9)
TZV	22.0 (9.7–44.0)
IPP on TRUS, mm	3.3 (0.0–7.4)
WBC, /μL	5880 (5000–6740)
NLR	1.93 (1.42–2.45)

Abbreviations: BMI, body mass index; PSA, prostate‐specific antigen; TRUS, transrectal ultrasound; TPV, total prostate volume; TZV, transitional zone volume; IPP, intravesical prostatic protrusion; WBC, white blood cell; NLR, neutrophil‐to‐lymphocyte ratio.

**Table 2:** Distribution of the NLR according to IPP in the overall cohort (n = 250)

	IPP <10 mm (n = 189)	IPP ≥10 mm (n = 61)	*P* value
Mean (SD)			
Age, y	64.0 (9.4)	69 (7.9)	0.019
BMI, kg/m^2^	24.0 (2.3)	24.7 (2.6)	0.085
PSA, ng/mL	2.5 (2.4)	4.7 (3.8)	<0.001
WBC, /μL	5930 (1070)	6028 (1180)	0.574
NLR	1.90 (0.7)	2.60 (1.2)	<0.001

Abbreviations: IPP, intravesical prostatic protrusion; SD, standard deviation; BMI, body mass index; PSA, prostate‐specific antigen; TRUS, transrectal ultrasound; WBC, white blood cell; NLR, neutrophil‐to‐lymphocyte ratio.

For further analyses, we used two cut‐off values: 40 cm^3^ for TPV and 10 mm for IPP (Table 3). Of the 250 patients, 142 had prostate volumes <40 cm^3^. In these 142 patients, we compared NLR between those with IPP < 10 mm (group 1) and those with IPP ≥10 mm (group 2), which revealed a significant difference (1.71 ± 0.57 vs 2.50 ± 0.71; *P* < 0.001). Similarly, in patients with larger volumes (≥40 cm^3^), a significant difference in NLR was noted between those with IPP < 10 mm (group 3) and those with IPP ≥10 mm (group 4) (2.07 ± 0.77 vs 2.50 ± 0.80; *P* < 0.001).

**Table 3:** Distribution of the NLR according to prostate volume and IPP

	TPV <40 cm^3^		TPV ≥40 cm^3^		*P* value
	IPP <10 mm (group 1, n = 120)	IPP ≥10 mm (group 2, n = 22)	IPP <10 mm (group 3, n = 69)	IPP ≥10 mm (group 4, n = 39)	
Mean (SD)					
Age, y	62.2 (6.6)	65.7 (5.8)	67.9 (9.4)	68.8 (9.0)	<0.001
BMI, kg/m^2^	24.2 (2.5)	24.1 (2.7)	23.6 (2.2)	24.5 (1.8)	0.779
PSA, ng/mL	1.4 (1.4)	. 2.1 (1.0)	4.1(3.4)	5.0 (3.9)	<0.001
WBC, /μL	5883 (1189)	6010 (987)	5931 (849)	6132 (1333)	0.769
NLR	1.71 (0.57)	2.50 (0.71)	2.07 (0.77)	2.50 (0.80)	<0.001

Abbreviations: TPV, total prostate volume; IPP, intravesical prostatic protrusion; SD, standard deviation; BMI, body mass index; PSA, prostate‐specific antigen; WBC, white blood cell; NLR, neutrophil‐to‐lymphocyte ratio.

We also compared NLR between the patients with TPV <40 cm^3^ and IPP ≥10 mm (n = 22, group 2) and those with TPV ≥40 cm^3^ and IPP <10 mm (n = 69, group 3), which again revealed a significant difference (2.50 ± 0.71 vs 2.07 ± 0.77; *P* = 0.020; Table 3).

Table 4 shows the result when we compared NLR between the patients with small (<40 cm^3^) prostates or prostates with IPP < 10 mm (n = 211) and those with larger prostates and IPP ≥10 mm (n = 39).

**Table 4:** Comparison of the NLR between small (<40 cm^3^) prostates or prostates with IPP <10 mm and larger prostates with IPP ≥10 mm

	Small (<40 cm^3^) prostate or prostate with IPP < 10 mm (n = 211)	Larger prostate with IPP ≥10 mm (n = 39)	*P* value
Mean (SD)			
Age, y	64.3 (9.2)	68.8 (9.0)	0.027
BMI, kg/m^2^	24.1 (2.4)	24.5 (1.8)	0.568
PSA, ng/mL	2.7 (1.9)	5.0 (3.9)	<0.001
WBC, /μL	5929 (1073)	6132 (1333)	0.778
NLR	1.99 (0.86)	2.50 (0.80)	<0.001

Abbreviations: IPP, intravesical prostatic protrusion; SD, standard deviation; BMI, body mass index; PSA, prostate‐specific antigen; WBC, white blood cell; NLR, neutrophil‐to‐lymphocyte ratio.

## 4 DISCUSSION

BPH involves the growth of epithelial and stromal cells in the transition zone and periurethral areas.^22^ The roles of androgens and growth factors in the onset and progression of BPH have been established over the last few decades, and inflammation has also been reported to play a role. Zlotta et al.^23^ demonstrated that chronic inflammation was noted in over 70% of men with BPH in their autopsy study, and there was an association between the degree of BPH and the level of chronic inflammation. Factors that may be associated with inflammation of the prostate involve infection, environmental or hormonal conditions, and systemic inflammation associated with metabolic syndrome.^3^, ^24^ Several reports have demonstrated the role of immune cell infiltration and pro‐inflammatory mediators in BPH pathogenesis.^25^, ^26^ For example, Nostrom et al.^25^ highlighted the involvement of interleukin‐8 and monocyte chemoattractant protein‐1.^25^ Chronic inflammation causes damage to prostatic tissue and subsequent repetitive process of wound healing; this tissue remodeling then results in the overgrowth of prostatic tissue.^1^, ^3^

NLR, a recognized surrogate marker of the state of inflammation in the body, is reasonable and can be simply determined from complete blood count by peripheral blood sampling. Its significance has been well highlighted in various medical fields.^7^, ^8^, ^9^, ^10^, ^11^, ^12^, ^13^ Several reports have demonstrated good correlations between NLR and severe LUTS or the progression of BPH.^3^ Tanik et al.^2^ reported that NLR was positively correlated with the International Prostate Symptom Score (IPSS) and negatively correlated with the maximum urinary flow rate (Q_max_) and the clinical status of patients. NLR showed stronger correlations with these parameters than erythrocyte sedimentation rate and C‐reactive protein. As for a reference value of NLR in the Korean population, Lee et al.^27^ investigated a large cohort (6268 men and 5892 women) of Koreans and reported that the mean NLR was 1.62, 1.67, and 1.73 for age groups 50s, 60s, and above 70s, respectively.

IPP results from the growth of prostatic lateral and median lobe. Several authors have reported that IPP might cause an obstruction of ‘ball valve’ type and malfunction of the funneling action by the bladder neck.^18^, ^28^ This protrusion has been reported to be significantly correlated with greater obstructive IPSS, decreased Q_max_, and increased postvoid residual urine volume.^17^, ^18^, ^20^ Thereafter, numerous studies have examined IPP and it has been reported to be a valuable anatomical marker for determining BOO which should be confirmed by urodynamic study.^17^ Lim et al.^20^ reported that IPP was a better predictor for BOO than was prostate volume, emphasizing that the existence of BOO is important to the urologists who can offer a more proactive treatment strategy such as surgery.

For these reasons, we focused in this study on the association between NLR and IPP. Although several studies^2^, ^3^ have reported associations between NLR and TPV (or TZV) or BPH progression, there was no report showing an association between NLR and IPP. The IPP threshold for defining BOO is recognized to be 10 mm, as was highlighted in a systematic review; this reported that IPP > 10 mm had a similar diagnostic accuracy as uroflowmetry alone.^29^ We therefore used the cut‐off value of 10 mm for IPP in the present study. NLR showed a significant correlation with IPP (Figure 1). In multivariate logistic regression analysis (Figure 2), NLR was found to be an independent predictor of IPP ≥10 mm. The cut‐off value for NLR predicting IPP ≥10 mm in our cohort was 2.2. A possible explanation of the correlation between NLR and IPP may be that chronic inflammation and the repetitive wound healing process results in a specific morphological change, intravesical protrusion of the prostatic median lobe.

Our analysis of patients with relatively small TPV (<40 cm^3^) showed that high NLR was associated with a high likelihood of having IPP ≥10 mm (group 1 vs group 2, Table 3). This unique group of patients having a small prostate gland with high IPP (and who would therefore be expected to be unobstructed according to TPV size criteria) is important because they experience obstruction during voiding due to the ‘ball valve’ effect caused by the IPP.^20^ As seen in Table 3, the difference in the NLR according to IPP was greater in the smaller prostate group than in the larger prostate group (1.71 vs 2.50 in group 1 vs 2 and 2.07 vs 2.50 in group 3 vs 4), although both had statistically significant differences.

We also compared NLR between the patients with small TPV (<40 cm^3^) and IPP ≥10 mm (group 2) and those with TPV ≥40 cm^3^ but with IPP < 10 mm (group 3). Interestingly, those with a small prostate and high IPP showed much higher NLR values than those with a larger prostate but without IPP (2.50 ± 0.71 vs 2.07 ± 0.77; *P* = 0.020, Table 3). This finding suggests that NLR is more closely related to the IPP of the median lobe than to TPV; however, a future study with a larger number of cases is needed to confirm this.

When we compared NLR between the patients with small (<40 cm^3^) prostates or prostates with IPP <10 mm and those with larger prostates and IPP ≥10 mm, those with a larger prostate and high IPP showed much higher NLR values than those with a smaller prostate or a prostate with IPP <10 mm (Table 4). This finding is in line with a previous report^3^ which demonstrated that more patients underwent surgical treatment when they have higher NLR, compared to those with lower NLR. The authors suggested that the standard medical therapy for BPH is not sufficient for patients with high prostatic inflammation. Similarly, Ficarra et al.^24^ reported that the use of alpha‐blockers (with or without 5‐alpha reductase inhibitors) can be insufficient to reduce LUTS in patients with high‐grade inflammation.

Based on the positive correlations between NLR and severe symptoms or the progression of BPH, several reports have proposed that anti‐inflammatory drugs could be used alongside standard medical therapy for BPH to prevent disease progression and relieve symptoms.^2^, ^3^ The use of traditional non‐steroidal anti‐inflammatory drugs (NSAIDs) and cyclooxygenase‐2 inhibitors has been proposed for relieving LUTS/BPH. A recent meta‐analysis of three randomized controlled trials that compared NSAIDs with placebo demonstrated that such drugs improved LUTS and uroflowmetric parameters.^30^ On this point, we agree with a previous report^3^ that demonstrated that the predictive value of NLR for treatment of BPH/LUTS may be more significant, although there has been no study yet analyzing the response to anti‐inflammatory drugs according to the serum NLR of the patient. The present study revealed a positive correlation between NLR and IPP. We think these findings provide additional data for future study to elucidate whether NLR can be used to determine who may benefit most from the anti‐inflammatory medication.

Although our study revealed a novel finding, the retrospective nature and relatively small number of patients of this investigation is the main limitation. Second, we were unable to clearly investigate the pathogenesis of the formation of IPP in patients with high NLR. Clearly, inflammation is not the only factor to cause IPP, and it is likely that various factors are associated with this morphological change. In particular, in men with normal NLR but high IPP, the IPP may have other causes than inflammation. On this issue, further studies of men with IPP at initial presentation are needed to determine whether the progression of IPP over time differs according to NLR. Nevertheless, we believe the present study provides adequate preliminary data with respect to the association between NLR and IPP. Finally, generalization of this study's results should be drawn with prudence, because all patients we studied were selected from a single institution in Korea.

In conclusion, our analysis demonstrated that NLR can be used as a surrogate marker for presence of a specific morphological change, IPP. The clinical value of NLR would be especially important in men with a small prostate gland but high IPP. The NLR was more strongly correlated with IPP than with TPV. Further studies are needed to confirm our findings.

## DISCLOSURE

The authors declare that they have no conflicts of interest related to the study.

## References

[1] Sebastianelli A , Gacci M . Current status of the relationship between metabolic syndrome and lower urinary tract symptoms. Eur Urol Focus. 2018;4:25‐27. 29602736 10.1016/j.euf.2018.03.007

[2] Tanik S , Albayrak S , Zengin K , et al. Is the neutrophil‐lymphocyte ratio an indicator of progression in patients with benign prostatic hyperplasia? Asian Pac J Cancer Prev. 2014;15:6375‐6379. 25124628 10.7314/apjcp.2014.15.15.6375

[3] Ozer K , Horsanali MO , Gorgel SN , Horsanali BO , Ozbek E . Association between benign prostatic hyperplasia and neutrophil‐lymphocyte ratio, an indicator of inflammation and metabolic syndrome. Urol Int. 2017;98:466‐471. 27464069 10.1159/000448289

[4] Gandaglia G , Briganti A , Gontero P , et al. The role of chronic prostatic inflammation in the pathogenesis and progression of benign prostatic hyperplasia (BPH). BJU Int. 2013;112:432‐441. 23650937 10.1111/bju.12118

[5] De Nunzio C , Kramer G , Marberger M , et al. The controversial relationship between benign prostatic hyperplasia and prostate cancer: the role of inflammation. Eur Urol. 2011;60:106‐117. 21497433 10.1016/j.eururo.2011.03.055

[6] Briganti A , Capitanio U , Suardi N , et al. Benign prostatic hyperplasia and its aetiologies. Eur Urol Suppl. 2009;8:865‐871.

[7] Walsh SR , Cook EJ , Goulder F , Justin TA , Keeling NJ . Neutrophil‐lymphocyte ratio as a prognostic factor in colorectal cancer. J Surg Oncol. 2005;91:181‐184. 16118772 10.1002/jso.20329

[8] Núñez J , Núñez E , Bodí V , et al. Usefulness of the neutrophil to lymphocyte ratio in predicting long‐term mortality in ST segment elevation myocardial infarction. Am J Cardiol. 2008;101:747‐752. 18328833 10.1016/j.amjcard.2007.11.004

[9] Tamhane UU , Aneja S , Montgomery D , Rogers EK , Eagle KA , Gurm HS . Association between admission neutrophil to lymphocyte ratio and outcomes in patients with acute coronary syndrome. Am J Cardiol. 2008;102:653‐657. 18773982 10.1016/j.amjcard.2008.05.006

[10] Halazun KJ , Hardy MA , Rana AA , et al. Negative impact of neutrophil‐lymphocyte ratio on outcome after liver transplantation for hepatocellular carcinoma. Ann Surg. 2009;250:141‐151. 19561458 10.1097/SLA.0b013e3181a77e59

[11] Celikbilek M , Dogan S , Ozbakır O , et al. Neutrophil‐lymphocyte ratio as a predictor of disease severity in ulcerative colitis. J Clin Lab Anal. 2013;27:72‐76. 23292894 10.1002/jcla.21564 PMC6807407

[12] Buyukkaya E , Karakas MF , Karakas E , et al. Correlation of neutrophil to lymphocyte ratio with the presence and severity of metabolic syndrome. Clin Appl Thromb Hemost. 2014;20:159‐163. 22992349 10.1177/1076029612459675

[13] Stotz M , Gerger A , Eisner F , et al. Increased neutrophil‐lymphocyte ratio is a poor prognostic factor in patients with primary operable and inoperable pancreatic cancer. Br J Cancer. 2013;109:416‐421. 23799847 10.1038/bjc.2013.332 PMC3721392

[14] Jang WS , Cho KS , Kim MS , et al. The prognostic significance of postoperative neutrophil‐to‐lymphocyte ratio after radical prostatectomy for localized prostate cancer. Oncotarget. 2017;8:11778‐11787. 28052031 10.18632/oncotarget.14349 PMC5355303

[15] Koo KC , Lee KS , Cho KS , Rha KH , Hong SJ , Chung BH . Comprehensive analysis and validation of contemporary survival prognosticators in Korean patients with metastatic renal cell carcinoma treated with targeted therapy: prognostic impact of pretreatment neutrophil‐to‐lymphocyte ratio. Int Urol Nephrol. 2016;48:985‐992. 26946137 10.1007/s11255-016-1252-9

[16] Lee KS , Ha JS , Koo KC . Significance of neutrophil‐to‐lymphocyte ratio as a novel indicator of spontaneous ureter stone passage. Yonsei Med J. 2017;58:988‐993. 28792143 10.3349/ymj.2017.58.5.988 PMC5552654

[17] Russo GI , Regis F , Spatafora P , et al. Association between metabolic syndrome and intravesical prostatic protrusion in patients with benign prostatic enlargement and lower urinary tract symptoms (MIPS Study). BJU Int. 2018;121:799‐804. 28872764 10.1111/bju.14007

[18] Chia SJ , Heng CT , Chan SP , Foo KT . Correlation of intravesical prostatic protrusion with bladder outlet obstruction. BJU Int. 2003;91:371‐374. 12603417 10.1046/j.1464-410x.2003.04088.x

[19] Lee SW , Cho JM , Kang JY , Yoo TK . Clinical and urodynamic significance of morphological differences in intravesical prostatic protrusion. Korean J Urol. 2010;51:694‐699. 21031089 10.4111/kju.2010.51.10.694 PMC2963782

[20] Lim KB , Ho H , Foo KT , Wong MY , Fook‐Chong S . Comparison of intravesical prostatic protrusion, prostate volume and serum prostatic‐specific antigen in the evaluation of bladder outlet obstruction. Int J Urol. 2006;13:1509‐1513. 17118026 10.1111/j.1442-2042.2006.01611.x

[21] St Sauver JL , Jacobson DJ , Girman CJ , McGree ME , Lieber MM , Jacobsen SJ . Correlations between longitudinal changes in transitional zone volume and measures of benign prostatic hyperplasia in a population‐based cohort. Eur Urol. 2006;50:105‐111. 16466847 10.1016/j.eururo.2006.01.013

[22] Roehrborn CG . BPH progression. Concept and key learning from MTOPS, ALTESS, COMBAT, and ALF‐ONE. BJU Int. 2008;101(Suppl 3):17‐21. 18307681 10.1111/j.1464-410X.2008.07497.x

[23] Zlotta AR , Egawa S , Pushkar D , et al. Prevalence of inflammation and benign prostatic hyperplasia on autopsy in Asian and Caucasian men. Eur Urol. 2014;66:619‐622. 25012523 10.1016/j.eururo.2014.06.026

[24] Ficarra V , Sekulovic S , Zattoni F , Zazzera M , Novara G . Why and how to evaluate chronic prostatic inflammation. Eur Urol Suppl. 2013;12:110‐115.

[25] Norström MM , Rådestad E , Sundberg B , et al. Progression of benign prostatic hyperplasia is associated with pro‐inflammatory mediators and chronic activation of prostate‐infiltrating lymphocytes. Oncotarget. 2016;7:23581‐23593. 26993768 10.18632/oncotarget.8051 PMC5029649

[26] Mazzucchelli L , Loetscher P , Kappeler A , et al. Monocyte chemoattractant protein‐1 gene expression in prostatic hyperplasia and prostate adenocarcinoma. Am J Pathol. 1996;149:501‐509. 8701989 PMC1865321

[27] Lee JS , Kim NY , Na SH , Youn YH , Shin CS . Reference values of neutrophil‐lymphocyte ratio, lymphocyte‐monocyte ratio, platelet‐lymphocyte ratio, and mean platelet volume in healthy adults in South Korea. Medicine (Baltimore). 2018;97:e11138. 29952958 10.1097/MD.0000000000011138 PMC6039688

[28] Suzuki T , Otsuka A , Ozono S . Combination of intravesical prostatic protrusion and resistive index is useful to predict bladder outlet obstruction in patients with lower urinary tract symptoms suggestive of benign prostatic hyperplasia. Int J Urol. 2016;23:929‐933. 27545297 10.1111/iju.13188

[29] Malde S , Nambiar AK , Umbach R , et al. Systematic review of the performance of noninvasive tests in diagnosing bladder outlet obstruction in men with lower urinary tract symptoms. Eur Urol. 2017;71:391‐402. 27687821 10.1016/j.eururo.2016.09.026

[30] Kahokehr A , Vather R , Nixon A , Hill AG . Non‐steroidal anti‐inflammatory drugs for lower urinary tract symptoms in benign prostatic hyperplasia: systematic review and metaanalysis of randomized controlled trials. BJU Int. 2013;111:304‐311. 23356748 10.1111/j.1464-410X.2012.11559.x

